# Quantitative methylation analyses of resection margins predict local recurrences and disease-specific deaths in patients with head and neck squamous cell carcinomas

**DOI:** 10.1038/sj.bjc.6604478

**Published:** 2008-07-01

**Authors:** H K Tan, P Saulnier, A Auperin, L Lacroix, O Casiraghi, F Janot, P Fouret, S Temam

**Affiliations:** 1 Department of Head and Neck Surgery, Institut Gustave-Roussy, 39, Rue Camille Desmoulins, Villejuif 94805, France; 2 Department of Surgical Oncology, National Cancer Center Singapore, 11 Hospital Drive, Singapore 169610, Singapore; 3 Translational Research Laboratory, Institut Gustave-Roussy, Rue Camille Desmoulins, Villejuif 94805, France; 4 Department of Biostatistics, Institut Gustave-Roussy, Rue Camille Desmoulins, Villejuif 94805, France; 5 Department of Pathology, Institut Gustave-Roussy, Rue Camille Desmoulins, Villejuif 94805, France; 6 Université Pierre et Marie Curie, 4 Place Jussieu, Paris 75005, France

**Keywords:** hypermethylation, margins, HNSCC, recurrence, QMSP, prognostic

## Abstract

This study sought to determine whether the presence of hypermethylated genes in the surgical margins can predict local recurrences in head and neck squamous cell carcinomas (HNSCCs). We prospectively collected tumour and surgical margin specimens from patients with HNSCCs who had undergone surgical resections. Quantitative methylation-specific PCR (QMSP) of CDKN2A, CCNA1 and DCC were performed in these specimens and correlated with clinical data. Of the 42 patients eligible for the study, 27 were hypermethylation informative for the above three genes. This latter group was associated with longer disease-free survivals (*P*=0.007) and longer time to disease-specific deaths (*P*=0.004). Multivariate analyses confirmed hypermethylation non-informative tumours as an independent prognosticating factor for disease-specific deaths (risk ratio 3.8, *P*=0.026). Quantitative MSP of the margins of 24 hypermethylation informative tumours revealed that 11 patients had molecularly positive margins, of which, five developed disease-specific events (DSEs, three local recurrences and two metastases), compared to none in patients with molecularly negative margins, after a median follow-up of 48 months. Log-rank analyses showed that molecularly positive margins were associated with shorter time to local recurrences and disease-specific deaths (*P*=0.03 and 0.01, respectively). This study demonstrated that QMSP of hypermethylated promoters in surgical margins predicted all the local recurrences in our series of HNSCC patients. We have also identified hypermethylation non-informative tumours as an independent predictor for the development of DSEs.

Head and neck squamous cell carcinoma (HNSCC) afflicts more than 500 000 patients per year (Parkin *et al*, 2005). Despite recent advances in therapeutic options, the mortality remains about 50% in 5 years. This is in part due to the high incidence of local recurrences (up to 50%) after surgery despite achieving histopathologically clear resection margins (Jesse and Sugarbaker, 1976; Davidson *et al*, 1981; Kowalski *et al*, 1993). Two possible mechanisms could account for these observations (Hockel and Dornhofer, 2005). First, minimal residual cancer cells might remain undetectable by standard histological margin assessment (Brennan *et al*, 1995; van Houten *et al*, 2004) and second, local recurrences may have evolved from a field of genetically altered mucosal cells (Slaughter *et al*, 1953; Califano *et al*, 1996; van Houten *et al*, 2004; Braakhuis *et al*, 2005; Martone *et al*, 2007). The detection of these ‘high risk’ lesions will allow clinicians to tailor adjuvant therapeutic options that can diminish the incidence of recurrences.

The improved understanding of tumour biology has uncovered many novel tumour markers that can distinguish between malignant and non-malignant cells (Sidransky, 2002). Brennan *et al*, (1995) first reported the use of *TP53* mutation for the detection of the residual tumour cells in surgical resection margins to predict locoregional recurrences. Various groups have also investigated surgical margins with other molecular markers such as eIF4E, PSA, tyrosinase and microsatellite instability to predict locoregional recurrences (Proebstle *et al*, 1996; Theodorescu *et al*, 1999; Sardi *et al*, 2000; Nathan *et al*, 2002; Temam *et al*, 2004).

More recently, silencing of tumour suppressor genes by hypermethylation of selected promoter regions has been identified as an important mechanism of carcinogenesis and implicated in a variety of solid and haematological malignancies (Laird and Jaenisch, 1994; Esteller *et al*, 2002; Momparler, 2003). As hypermethylated promoters often occur from 100- to 1000-folds more frequently in tumour cells compared to normal cells, they are therefore good candidate molecular markers for detection of low numbers of tumour cells in the milieu of normal cells (Laird, 2003). Methylation-specific PCR (MSP) and quantitative MSP (QMSP) have been used to detect methylated DNA in the body fluids of patients with cancers of the breast (Silva *et al*, 1999, 2002), lung (Esteller *et al*, 1999), prostate (Goessl *et al*, 2000), bladder (Dominguez *et al*, 2002), gastrointestinal tract (Kawakami *et al*, 2000; Eads *et al*, 2001; Zou *et al*, 2002) and head and neck region (Sanchez-Cespedes *et al*, 2000; Righini *et al*, 2007). There are however few reports of methylation analyses of resection margins. Goldenberg *et al*, (2004) demonstrated in a pilot study with six patients that intra-operative assessments of surgical margins methylation status were feasible using QMSP. No recurrence was observed in any of the three patients with positive molecular resection margin but the follow-up period was short. Shaw *et al*, (2007b) performed quantitative methylation analyses of resection margins in 20 patients using pyrosequencing methylation analyses (PMA) . However, 6 out of 13 positive margins subsequently developed locoregional recurrences, there were however two recurrences in seven negative margins. The authors suggested that the small sample size and the use of fixed tissue DNAs may have contributed to the inconclusive findings. Furthermore, the use of pyrosequencing with a sensitivity of 1 out of 20 to 1 out of 50 may not be appropriate for detection of small numbers of tumour cells in surgical margins. Most recently, Martone *et al*, (2007) identified similar methylation profiles between tumours and margins in 9 out of 11 patients with HNSCC-specific deaths. As this study consisted only of retrospectively selected patients who had relapses and HNSCC-specific deaths but no disease-free controls, it did not identify significant association between methylation statuses in surgical margins with disease-free survivals.

The objective of our study was to measure the amount of hypermethylated promoters of selected genes, in a series of prospectively collected surgical margins of HNSCC patients, to predict local recurrences and cancer-specific survivals. We utilised QMSP to interrogate the methylation profiles of CDKN2A, CCNA1 and DCC in the resection margins in a cohort of patients who underwent curative resections of HNSCCs. We selected these genes on the basis of reports that CDKN2A (Shaw *et al*, 2006), CCNA1 (Shaw *et al*, 2006) and DCC (Carvalho *et al*, 2006) are hypermethylated in 28, 53 and 75% of HNSCCs respectively. Significantly, all three genes have very low level of methylation in normal mucosa and are thus good candidate genes for the purpose of our study. Functionally, CDKN2A (Kim and Sharpless, 2006) and DCC (Mehlen and Fearon, 2004; Carvalho *et al*, 2006) are known tumour suppressor genes, whereas CCNA1 (Ji *et al*, 2005) has been shown to play a role in cell cycle regulation. The study was conducted in two phases: (1) to determine the hypermethylation informative statuses of the primary tumours and (2) to detect the presence of promoter hypermethylation in the surgical margins of those tumours that were hypermethylation informative; all findings were then correlated with clinical data.

## Materials and methods

### Sample collection and histopathological examination

We prospectively collected fresh frozen tumour biopsies and surgical margin specimens from patients treated for HNSCCs at Institut Gustave-Roussy (Villejuif, France) between January and December 2000. The Institutional Review Board approved the study, and informed consent was obtained from all patients. Tumour staging (TNM and pTNM) was performed according to the Union International Contre Cancer 1997 criteria. The study design, samples collection and histopathological examinations of surgical specimens were performed as previously described (Temam *et al*, 2004).

Patients with close resection margins (⩽5 mm) on surgical specimens or moderate-to-severe dysplasia on surgical margins were excluded. To ensure adequate representation of the margins, patients with less than three margins available were excluded from the second phase of the study.

During the study period, 42 patients met the inclusion criteria and were recruited for the study. All patients underwent as first treatment surgical resection of the primary tumours and unilateral or bilateral neck dissections depending on tumour sites and nodal status. The indication for postoperative radiotherapy depended on tumour stage, tumour site and nodal status.

### DNA extraction and bisulphite treatment

All tumour biopsy specimens included in the analysis were diagnosed as invasive HNSCCs. Serial 40-*μ*m thick frozen sections of each tumour or surgical margin specimen were performed with H&E staining for histopathologic control of the first and the last 5-*μ*m thick section. A pathologist (PF), blinded to the clinical data, reviewed all the slides to confirm (1) the percentage of tumour cells present in the tumour biopsies or (2) the absence of carcinomas or moderate-to-severe dysplasias in each surgical margin.

DNA from tumour and surgical margin specimens was extracted using the QIAamp Tissue kit (Qiagen, Courtaboeuf, France). DNA quality was verified using GeneQuant II (Amersham Pharmacia Biotech, Cambridge, UK).

The extracted DNA was modified by sodium bisulphite in accordance to the manufacturer's protocol (Chemicon no. 7280) and resuspended in 100 *μ*l of TE buffer (EDTA 2.5 mmol l^−1^ and Tris-HCl 10 mmol l^−1^) and stored at −20°C.

### Methylation analysis

The bisulphite-modified DNA was used as a template for QMSP, as previously described (Harden *et al*, 2003) with some modifications. In brief, the primers and probes were designed to specifically amplify the bisulphite-converted promoter of the gene of interest. The primers and probe sequences for CDKN2A and *β-*actin have been previously described (Lo *et al*, 1999; Harden *et al*, 2003). The sequences of CCNA1 and DCC are listed below in the order of forward primer, reverse primer and probe: CCNA1; 5′-GCGGTTTCGGAGAGCGTAC-3′, 5′-GACGCCCCCGAACCTAAC-3′, 6FAM5′-TTTGTCGCGGTCGGTATGGAAACG-3′TAMRA, DCC; 5′-TGTTCGCGATTTTTGGTTTC-3′, 5′-ACCGATTACTTAAAAATACGCG-3′, 6FAM5′-TTTTCGGAGTTTTTTTGTTTAGCGC-3′TAMRA.

Fluorogenic PCRs were carried out in a reaction volume of 50 *μ*l consisting of 300 nM of each primer; 150 nM of probe. 10 *μ*l of treated DNA solution were used in each real-time MSP reaction. Amplifications were carried out in 96-well plates in a 7900 Sequence Detector System (Perkin-Elmer Applied Biosystems, Norwalk, CT, USA). Commercially available methylated DNA (CpGenome Universal Methylated DNA AbCys S.A. no. S7821) were used as positive controls and serial dilutions of this DNA were used for constructing the calibration curves to detect between five genomic equivalents and 15 000 genomic equivalents of DNA on each plate. Identical laboratory procedures and intermixing were performed in the same laboratory for each batch tested. All tumours and margins DNA for each test batch were bisulphite modified at the same time with their positive and negative controls to minimise variations in experimental conditions. All plates contained multiple water blanks and bisulphite modified Human Genomic DNA (CpGenome Universal Unmethylated DNA no. S7822) as negative controls. Every QMSP was performed in duplicates or triplicates.

### Calculation of methylation index and weighted tumour methylation index

Methylation index (MI) was computed as the ratios between the methylation values of the gene of interest and the internal reference gene, *β-*actin, which were obtained by Taqman analysis (Harden *et al*, 2003). These ratios were used as measures to represent the relative levels of methylation in any given sample:

MI=gene of interest methylation value/*β*-actin methylation value × 100%.

Given that tumour specimens comprised between 50 and 100% of malignant cells, the MI obtained from tumour specimens therefore represented the average methylation levels of a mixture of the normal and malignant cells. As methylation of CDKN2A, CCNA1 and DCC exist only in very low levels in normal cells (Carvalho *et al*, 2006; Shaw *et al*, 2006), we calculated a separate index, which we termed as the weighted tumour methylation index (WTMI) to better reflect methylation levels in the tumour cells:

Weighted tumour methylation index (WTMI)=MI of the tumour specimens divided by the percentage of malignant cells present in the tumour specimens (MI/percentage of tumour × 100%).

#### Stratification of tumours and margins

In contrast to the semiquantitative nature of MSP, QMSP generates precise MI in continuous variables and thus necessitates that a threshold be set to dichotomise the data. We employed two thresholds to stratify (a) tumours into hypermethylation informative or non-informative using WTMI of the tumour specimens and (b) margins into molecularly positive or negative using MI of the margin specimens:

*Stratification of tumours*. Tumours were assigned to be hypermethylation informative only if WTMI are ⩾5%. The cutoff at 5% was chosen to select only tumours that have very high levels of hypermethylation to allow discriminative detection of very small numbers of informative tumour cells mixed in a large numbers of normal cells in the surgical margins.

*Stratification of margins*. Quantitative MSP for the margins were performed only on the tumours that were hypermethylation informative and only on the genes that the tumours were informative for. We then ranked the margins in ascending order according to the MI obtained for each of the gene tested. The margins with MI above the 90th percentile of all the margins tested for that gene were deemed molecularly positive for that corresponding gene. This threshold was set to allow exclusions of margins with low levels of MI of unknown significance.

### Statistical analysis

This prospective study was powered based on a previous study conducted by one of our co-authors (Temam *et al*, 2004), which demonstrated an disease-free survival (DFS) at 30 months of 85% among patients with negative margins and 30% among patients with positive margins (using microsatellite instability as markers). To achieve a study with 80% power, we calculated prospectively that 22 informative patients were required to demonstrate a similar difference in DFS at 30 months with an *α*-risk of 5%, working on the basis that half of the informative patients have positive margins for hypermethylation in our study. Univariate analyses using Fisher's exact test and Student's *t*-test were performed for categorical and continuous data, respectively. Clinical and biological characteristics were analysed for their association with time to local recurrence using Cox proportional hazards models. Estimates of survival curves were calculated according to the Kaplan–Meier product-limit method and were calculated from the time of surgery to the time of death or the last follow-up visit. Times to local recurrence for various prognostic groups were compared using the log-rank test. The Cox proportional hazards regression model was used to assess the prognostic effect of patient characteristics and molecular markers to estimate DFS and time to disease-specific death. Predictive variables with *P*-values of ⩽0.10 for the univariate Cox proportional hazards model were included in a multicovariate model. All of the computations carried out were performed using NCSS and SPSS software.

## Result

### Methylation profiles of tumours

**Figure 1:**
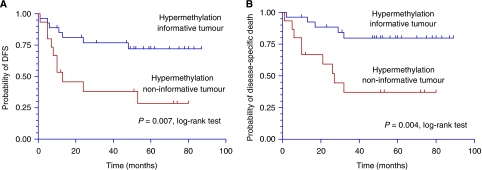
Kaplan–Meier curve of hypermethylation informative tumour *vs* hypermethylation non-informative tumour in (**A**) disease-free survival and (**B**) time to disease-specific death.

Quantitative MSP of the three selected genes CDKN2A, CCNA1 and DCC was performed on 42 tumours. High levels of hypermethylation (WTMI ⩾5%, range 5.1–95%) were detected in one or more of the three genes in 64.3% (*n*=27/42) of tumours. These were designated as hypermethylation informative as described (Materials and Methods). Baseline clinical characteristics such as age, sex, site, stage, histopathological gravity signs and adjuvant therapy did not differ significantly according to the tumour methylation profiles (Table 1). The median clinical follow-up period was 48 months (range, 1–89). Only three patients were lost to follow-up within 24 months of diagnosis. Mean follow-up period for patients who remained events free were 70 months. Sixteen patients developed one or more disease-specific events (DSEs, defined as local and/or regional recurrences and/or distant metastasis) with median DFS of 12.25 months (range, 1–53). The hypermethylation informative group fared better, compared to the non-informative group; with longer DFSs and longer time to disease-specific deaths (*P*=0.007 and 0.004, respectively, Kaplan–Meier analysis with log-rank test) as shown in Figure 1. Multi-covariate analyses with possible confounding factors such as age, sex, site, stage, histopathological gravity signs and adjuvant therapy identified hypermethylation non-informative tumours as an independent prognosticating factor for DFS with a risk ratio of 3.82 (Table 2).

### Molecular analyses of surgical margins

In the second phase of this study, we performed QMSP on the resection margins of the hypermethylation informative tumours. Three patients with informative tumours were excluded from this phase because there were less than three margins available for analysis (Materials and Methods). One hundred-thirteen margins from the remaining 24 patients (3–7 margins per patient) underwent QMSP analyses for the genes that were informative in the primary tumours. The majority of the margins had very low methylation levels for CDKN2A, CCNA1 and DCC, with median MI at 0.08% (range 0–0.39%), 0.06% (range 0–31.9%) and 0.08% (range 0–2.28%), respectively. The threshold MI levels for defining margins molecular positivity, set at 90th percentile for each of the gene tested, were 0.36% (CDKN2A), 0.87% (CCNA1) and 0.91% (DCC). Twelve margins from 11 patients were deemed molecularly positive; 10 patients had one margin that were positive for one or more gene tested, whereas one other patient had two margins that were positive for the same gene.

Baseline clinical characteristics did not differ according to margins methylation status (Table 3). Five of the 11 patients with molecularly positive surgical margins developed DSE (three local recurrence, two distant metastases) compared to none in the 13 patients with molecularly negative surgical margins (*P*=0.011, Fisher's exact test).

The local recurrences occurred in two oropharyngeal cancers (stage IV) and one oral cavity cancer (stage II). The distant metastases were both lung metastases and had occurred in one oral cavity cancer (stage II) and one oropharyngeal cancer (stage IV).

**Figure 2:**
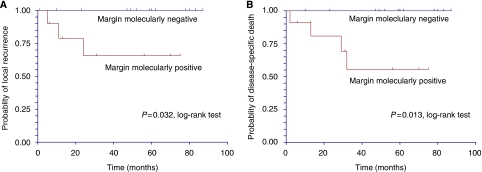
Kaplan–Meier curves of margin molecularly positive *vs* margin molecularly negative in (**A**) time to local recurrence and (**B**) time to disease-specific death.

Molecular positivity in surgical margins were significantly associated with decreased time to local recurrences (*P*=0.03, Kaplan–Meier analysis with log-rank test, Figure 2A). The latter was not significantly associated with other covariates (age, sex, site, stage, histopathological gravity signs and adjuvant therapy) by Cox regression analysis (not shown). Furthermore, molecularly positive surgical margins were associated with decreased time to disease-specific deaths (*P*=0.013, Kaplan–Meier analysis with log-rank test, Figure 2B).

## Discussion

The objective of our study was to measure the relative amount of hypermethylated promoters of selected genes in the surgical margins of HNSCC patients and to determine whether these could predict local recurrences and cancer-specific survivals. The most important finding in our study is that the molecularly positive margins identified by QMSP correctly predicted all five DSE (three local recurrences and two distant metastases), whereas none of the patients with molecularly negative margins developed any DSE at a median follow-up period of 48 months.

### WTMI and prognosis

Sixty-four per cent of the HNSCCs in our cohort of patients were informative for hypermethylation. This compares favourably with the ratio of informative tumour using other molecular markers such as *TP53* mutation (50% informative) (Boyle *et al*, 1993; Brennan *et al*, 1995) and MSI (48% informative) (Temam *et al*, 2004). We noted that tumour stage was not associated with recurrence in this series, probably because we selected patients after exhaustive pathological assessments or because the sample size was small for subset analyses.

Our findings that patients with hypermethylated tumours have better prognosis corroborated with recent reports of others. A recent study reported promoter methylation profiles of hMLH1, MGMT and CDKN2A in 51 cases of HNSCCs using MSP and demonstrated that tumours with two or more methylated genes had improved DFS at 2 years (Puri *et al*, 2005). Koscielny *et al*, (2007) reported in his series of 67 patients that hypermethylation and LOH were the two main mechanisms responsible for inactivating CDKN2A but only the hypermethylated group were associated with a lower incidence of locoregional recurrence. Shaw *et al*, (2007a) observed in a series of 76 patients that tumours with CpG island methylation phenotype (CIMP) were associated with marked inflammatory response and less aggressive tumour biology. These reports and our findings in this study are emerging evidence that hypermethylation of certain genes in HNSCCs may be associated with longer survival. Unfortunately, available literature does not provide clear insight to the mechanism behind this counter-intuitive observation that hypermethylation of tumour suppressor genes in HNSCCs can lead to better prognosis. Nevertheless, a possible parallel mechanism can be drawn from studies in colorectal cancers. There is convincing evidence that CIMP and chromosomal instability represent two independent and inversely related mechanisms of genetic and epigenetic instability in sporadic colorectal carcinomas (Goel *et al*, 2007). Furthermore, CIMP underlies a subtype of colorectal cancer with microsatellite instability with distinctive clinical features (Ribic *et al*, 2003; Weisenberger *et al*, 2006). We postulate that a similar mechanism may be at work in HNSCCs, where hypermethylated tumours constitute a subgroup of HNSCCs with a distinctive clinical profile.

The possible presence of LOH in CDKN2A (Koscielny *et al*, 2007) and DCC (Papadimitrakopoulou *et al*, 1998) may increase the likelihood of tumours being non-informative for these two markers. However, this is unlikely to confound our findings in the margin analyses of informative tumours. Furthermore, the absence of data that clearly outline the interaction between LOH and hypermethylation has made it difficult to adjust our scoring system to cater for the presence of LOH.

### MI of surgical margins and recurrence

All three local recurrences and two distant metastases occurred in the 11 (out of 24) patients with molecularly positive margins. We noted that 7 out of the 11 patients with molecularly positive margins underwent adjuvant radiotherapy and, of these, only one patient developed local recurrence. In contrast, of the remaining four patients who did not have adjuvant radiotherapy therapy, two had developed local recurrences. It is likely that adjuvant treatments have prevented local recurrences that might have otherwise occurred in some of these patients with molecularly positive margins and thus may partly account for the ‘false-positive’ patients.

In contrast, all 13 patients with molecularly negative margins turned out to be true negative. We noted that four of these were hypopharyngeal tumours (often associated with the poorest prognosis), while there was none in the group with positive margins. Although this disparity in tumour sites did not reach statistical significance (*P*=0.067, Fisher's exact test), probably due to the small sample size, we were nonetheless reassured that the specificity of the molecularly negative margins could not be attributed to the selection of tumour sites with favourable prognosis.

### Implication

Our findings have both clinical and scientific implications to HNSCCs. Clinically, the poorer prognosis of patients with non-informative tumours or molecularly positive surgical margins may warrant either closer follow-up or recommendation of adjuvant therapy, even in the absence of other risk factors such as advanced stage, perineural invasion and perivascular invasion. Conversely, in cases where the benefit of adjuvant therapy is equivocal, methylation profiles of the tumours or the margins can serve as additional indicators to tailor sensible treatment strategies so as to minimise unnecessary treatment morbidities. However, these results must be verified in an independent prospective study before it can be used to formulate clinical guidelines. We are in the process of incorporating such a validation study into an ongoing international, multicentric, prospective controlled trial led by Institute Gustave-Roussy.

For any tumour molecular marker to be clinically relevant, it should ideally be informative in the majority of that specific tumour. In previous reports of similarly designed studies, molecular markers such as *TP53* and *MSI* were both informative in about 50% of HNSCCs. In comparison, hypermethylation was informative in almost two-thirds of the HNSCCs using our panel of three genes, even though we only designated tumours with very high level of hypermethylation as informative. The percentage of informative tumours can conceivably be further increased by screening other suitable candidate genes. Hypermethylated genes are therefore good molecular markers for HNSCCs in the clinical settings.

Scientifically, our findings together with other reports would suggest that hypermethylation of certain gene loci may confer a tumour phenotype with distinctive clinical characteristics. Further research is warranted to delineate the exact roles of hypermethylation and other alternative mechanisms such as genetic deletion and point mutation in HNSCC carcinogenesis.

This study also illustrates that QMSP is ideally suited for detection of hypermethylated markers in surgical margins. It detects promoter hypermethylation with great sensitivity and specificity. In addition, it is technically robust and easily reproducible. Most importantly, it offers significant advantage to conventional MSP because of its ability to generate accurate quantitative data as a continuous variable. The latter allows the dichotomy of data at a threshold to facilitate stratification, a feature that we leverage on to determine the tumour methylation profile (hypermethylation informative *vs* non-informative) and the margin methylation status (molecularly positive *vs* negative).

### Summary

This is to our knowledge the first study to report that presence of hypermethylated promoters in surgical margins of HNSCCs can predict local recurrences and disease-specific deaths. Performing QMSP with a panel of three genes (CDKN2A, CCNA1 and DCC), we found 64% of HNSCC tumours were hypermethylation informative. Methylation analyses of their resection margins correctly predicted all the recurrences in this cohort of patients. Furthermore, we have showed that HNSCCs with hypermethylation of these three gene promoters have a better prognosis.

## Figures and Tables

**Table 1:** Patient characteristics according to tumour methylation status

	**Tumour hypermethylation**		
	**Informative (*n*=27)^a^**	**Non-informative (*n*=15)**	***P*-value^b^**
*Age, years*			
Mean±s.d.	59.67±9.7	57.07±10.46	
Median (maximum, minimum)	61 (32, 77)	61 (39, 73)	0.72
			
*Sex,* n (%)			
Male	22 (81.5)	12 (80)	
Female	5 (18.5)	3 (20)	1
			
*Site,* n (%)			
Oral cavity	10 (37.0)	6 (40.0)	
Oropharynx	10 (37.0)	2 (13.3)	
Hypopharynx	5 (18.5)	3 (20.0)	
Larynx	2 (7.4)	4 (26.7)	0.218
			
*Tumour stage,* n (%)			
I–II	8 (29.6)	2 (13.3)	
III–IV	19 (70.4)	13 (86.7)	0.286
			
*Histopathological gravity signs*^c^, n (%)			
No	24 (88.9)	10 (66.7)	
Yes	3 (11.1)	5 (33.3)	0.11
			
*Adjuvant therapy*^d^, n (%)			
No	14 (51.9)	12 (80.0)	
Yes	13 (48.1)	3 (20.0)	0.102
			
*Disease-specific events*,n (%)			
No	20 (74.1)	6 (40.0)	
Yes	7 (25.9)	9 (60.0)	0.047
			
*Disease-specific death*,n (%)			
No	22 (81.5)	6 (40.0)	
Yes	5 (18.5)	9 (60.0)	0.015

a Informative: high level of gene promotor hypermethylation (WTMI ⩾ 5%).

b Fisher's exact test.

c Histopathological gravity signs: perineural invasion, angiolymphatic invasion or both.

d Concurrent chemoradiotherapy or radiotherapy alone.

**Table 2:** Univariate and multivariate Cox proportional hazards models in estimating time to disease-specific death

**Covariates**	**Regression coefficient**	**s.e.**	**Risk ratio**	**Probability**
Age, years	−0.014	0.025	0.986	0.567
Sex (male *vs* female)	0.813	0.758	2.254	0.284
Stage (I–II *vs* III–IV)	0.012	0.667	1.012	0.985
Adjuvant therapy^a^ (yes *vs* no)	0.306	0.548	1.358	0.577
Histopathological gravity sign (yes *vs* no)	0.377	0.602	1.458	0.531
Hypermethylation informative (− *vs* +)	1.341	0.602	3.823	0.026^b^

a Concurrent chemoradiotherapy or radiotherapy alone.

b No covariate other than tumour methylation status was entered in the multivariate analyses, because *P*>0.10 for all other covariates.

**Table 3:** Patient characteristics according to margin methylation status

	**Margin hypermethylation**		
	+	−	***P*-value^a^**
*Age, years*			
Mean±s.d.	61.6±11.1	58.3±9.7	
Median (maximum, minimum)	64 (40, 77)	61 (32, 68)	0.72
			
*Sex*			
Male	8 (72.7)	12 (92.3)	
Female	3 (27.3)	1 (7.7)	0.3
			
*Site,* n (%)			
Oral cavity	6 (54.5)	4 (30.8)	
Oropharynx	5 (45.5)	4 (30.8)	
Hypopharynx	0 (0)	4 (30.8)	
Larynx	0 (0)	1 (7.7)	0.15
			
*Tumour stage,* n (%)			
I–II	4 (36.4)	3 (23.1)	
III–IV	7 (63.4)	10 (76.9)	0.67
			
*Histopathological gravity signs*^b^, n (%)			
No	9 (81.8)	12 (92.3)	
Yes	2 (18.2)	1 (7.7)	0.58
			
*Adjuvant therapy*^c^, n (%)			
No	4 (36.4)	7 (53.8)	
Yes	7 (63.4)	6 (46.2)	0.17
			
*Disease-specific event,* n (%)			
No	5 (45.5)	13 (100)	
Yes	6 (54.5)	0 (0)	0.01
			
*Locoregional recurrence,* n (%)			
No	8 (72.7)	13 (100)	
Yes	3 (27.3)	0 (0)	0.03^d^
			
*Disease-specific death,* n (%)			
No	7 (63.4)	13 (100)	
Yes	4 (36.4)	0 (0)	0.01^d^

a Fisher's exact test.

b Histopathological gravity signs: perineural invasion, angiolymphatic invasion or both.

c Concurrent chemoradiotherapy or radiotherapy alone.

d Log-rank test.
